# Insulin sensitivity estimates and their longitudinal association with coronary artery disease in type 1 diabetes. Does it matter?

**DOI:** 10.1186/s12933-024-02234-x

**Published:** 2024-05-03

**Authors:** Stefan Mutter, Erika B. Parente, Andrzej S. Januszewski, Johan R. Simonsen, Valma Harjutsalo, Per-Henrik Groop, Alicia J. Jenkins, Lena M. Thorn, the FinnDiane Study Group

**Affiliations:** 1 Folkhälsan Institute of Genetics, Folkhälsan Research Center, Biomedicum Helsinki, Haartmaninkatu 8, 00290 Helsinki, Finland; 2 Department of Nephrology, University of Helsinki and Helsinki University Hospital, Haartmaninkatu 4, 00290 Helsinki, Finland; 3 Research Program for Clinical and Molecular Metabolism, Faculty of Medicine, University of Helsinki, Haartmaninkatu 8, 00290 Helsinki, Finland; 4 Sydney Pharmacy School, University of Sydney, A15, Science Rd, Camperdown, NSW 2050 Australia; 5 NHMRC Clinical Trials Centre, University of Sydney, K25, Parramatta Rd, Camperdown, NSW 2050 Australia; 6 Department of Diabetes, Central Clinical School, Monash University, The Alfred Centre, 99 Commercial Rd, Melbourne, VIC 3004 Australia; 7 Baker Heart and Diabetes Institute, 75 Commercial Rd, Melbourne, VIC 3004 Australia; 8 Department of General Practice and Primary Health Care, University of Helsinki and Helsinki University Hospital, Biomedicum 2, Tukholmankatu 8, 00290 Helsinki, Finland

**Keywords:** Insulin resistance, Type 1 diabetes mellitus, Coronary artery disease, Cardiovascular diseases, Kidney disease

## Abstract

**Background:**

Insulin resistance and chronic kidney disease are both associated with increased coronary artery disease risk. Many formulae estimating glucose disposal rate in type 1 diabetes infer insulin sensitivity from clinical data. We compare associations and performance relative to traditional risk factors and kidney disease severity between three formulae estimating the glucose disposal rate and coronary artery disease in people with type 1 diabetes.

**Methods:**

The baseline glucose disposal rate was estimated by three (Williams, Duca, and Januszewski) formulae in FinnDiane Study participants and related to subsequent incidence of coronary artery disease, by baseline kidney status.

**Results:**

In 3517 adults with type 1 diabetes, during median (IQR) 19.3 (14.6, 21.4) years, 539 (15.3%) experienced a coronary artery disease event, with higher rates with worsening baseline kidney status. Correlations between the three formulae estimating the glucose disposal rate were weak, but the lowest quartile of each formula was associated with higher incidence of coronary artery disease. Importantly, only the glucose disposal rate estimation by Williams showed a linear association with coronary artery disease risk in all analyses. Of the three formulae, Williams was the strongest predictor of coronary artery disease. Only age and diabetes duration were stronger predictors. The strength of associations between estimated glucose disposal rate and CAD incidence varied by formula and kidney status.

**Conclusions:**

In type 1 diabetes, estimated glucose disposal rates are associated with subsequent coronary artery disease, modulated by kidney disease severity. Future research is merited regarding the clinical usefulness of estimating the glucose disposal rate as a coronary artery disease risk factor and potential therapeutic target.

**Supplementary Information:**

The online version contains supplementary material available at 10.1186/s12933-024-02234-x.

## Background

Insulin resistance, commonly observed in type 2 diabetes, can also occur in people with type 1 diabetes [1]. This is sometimes referred to as ‘double diabetes’, and is characterised by the presence of components of the metabolic syndrome, such as adiposity, hyperglycaemia, hypertension, and dyslipidaemia [2], and is associated with a higher risk of micro- and macrovascular complications and death [3–6]. A definition of the metabolic syndrome has been agreed upon by an international consensus group, but notably, this definition is not specific for people with type 1 diabetes [7]. Whilst the gold standard method of quantifying insulin sensitivity, the euglycaemic hyperinsulinaemic clamp [8] is not feasible in large studies or in clinical practice. Many formulae using clinically available variables have been developed to estimate insulin sensitivity, such as the estimated glucose disposal rate (eGDR) [9–13]. These equations have been derived from clamp studies in several dozens of people with type 1 diabetes, usually adults, without or with only a few with chronic complications, potentially limiting generalisability. Furthermore, validation of eGDR calculated from these formulae and GDR measured in independent clamp studies showed rather weak correlations, with r < 0.3 [10, 11, 14]. Nevertheless, eGDR calculated from some formulae have been shown to be associated with subsequent diabetic kidney disease [4, 15, 16], diabetic retinopathy [4], cardiovascular disease [15, 17], and mortality [18] in people with type 1 diabetes. We are not aware of any publications that have evaluated more than one eGDR formulae in relation to subsequent chronic diabetes complications, nor evaluated eGDR performance according to diabetic kidney disease status.

We aim, thus, to compare three eGDR formulae [10–12] that are based on the three available clamp studies performed in adult Caucasians with type 1 diabetes, and assess their relationship with subsequent incidence of coronary artery disease (CAD) over a long follow-up in a large cohort of adults with type 1 diabetes, including evaluating eGDR formulae performance according to baseline kidney disease severity.

## Methods

### Study participants

All participants are from the FinnDiane Study, an ongoing, multicentre, observational, Finnish study founded in 1997, including 77 study centres (Additional file 1: Table S1), which aims to discover clinical, genetic, and environmental risk factors for micro- and macro-vascular complications of type 1 diabetes [2]. We included 3517 participants with type 1 diabetes, defined as age of diabetes onset < 40 years and insulin treatment from within 1-year of diagnosis who had their baseline visit before the end of 2017 (median 2000, IQR 1998, 2002). We excluded individuals with pre-existing (pre-baseline) cardiovascular events, including myocardial infarction, coronary revascularisation, stroke, amputations, or peripheral artery revascularisation, as well as those with kidney replacement therapy or an estimated glomerular filtration rate (eGFR) < 15 ml/min/1.73 m^2^. We further excluded individuals with missing data required for eGDR calculation by the three formulae of interest.

Baseline clinical characteristics were as previously described [2]. *Anthropometrics*: weight in light clothing to the closest 0.1 kg, height to the closest one cm, waist circumference midway between lowest ribs and iliac crest and hip circumference at widest part of gluteal region, both to closest 0.5 cm; *blood pressure (BP)*: the mean of two measures of systolic and diastolic BP taken seated after ten minutes rest; *medical history*: including history of diabetes and its complications, current medications, insulin pump use, and lifestyle by validated questionnaires; *clinical chemistry*: venous blood for creatinine, HbA_1c_, lipids, and lipoproteins; *kidney status*: eGFR calculated by chronic kidney disease epidemiology collaboration equation [19], albuminuria status based on albumin excretion rate (AER) in two out of three urine collections classified as: normal AER (< 20 µg/min or < 30 mg/24 h), moderately increased albuminuria (20–200 µg/min or 30–300 mg/24 h), or severely increased albuminuria (> 200 µg/min or > 300 mg/24 h). Diabetic kidney disease severity as per Kidney Disease: Improving Global Outcomes (KDIGO) risk categories: low risk (normal AER and eGFR ≥ 60 ml/min/1.73 m^2^, N = 2513), moderately increased risk (normal AER and eGFR 45–59 ml/min/1.73 m^2^, or moderately increased albuminuria and eGFR ≥ 60 ml/min/1.73 m^2^, N = 516), as well as high (N = 251) and very high risk (N = 237) (normal AER and eGFR < 45 ml/min/1.73 m^2^, moderately increased albuminuria and eGFR < 60 ml/min/1.73 m^2^, or severely increased albuminuria irrespective of eGFR) [20]. Due to relatively low numbers, the high and very high KDIGO risk groups were combined.

### eGDR and the metabolic syndrome

eGDR was estimated by three formulae for adult Caucasians:

1. Modified Williams formula: The original formula [12], modified for use of HbA_1c_ vs. HbA_1_ [2]. eGDR = 24.4−(12.97*WHR)-(3.39*AHT)−(0.60*HbA_1c_ [%]), where WHR = waist-to-hip ratio and AHT = antihypertensive treatment and/or BP ≥ 140/90 mmHg (yes = 1; no = 0).
2. Duca formula: The best fit formula without adiponectin by Duca et al. [10]: eGDR = exp[4.1075-(0.01299*waist circumference [cm])-(1.05819*daily insulin dose per body weight [IU/kg]−(0.31327*triglycerides [mmol/L])−(0.00802*diastolic BP [mmHg]).
3. Januszewski formula [11]: eGDR = 6.6743 + (6.1818*sex [Woman = 0; Man = 1]) + (0.0708*age [years]) + (7.4104*HDL cholesterol [mmol/L])−(0.1692*pulse pressure [mmHg])−(0.0894*serum creatinine [µmol/L]).

Insulin resistance was defined as the lowest quartile of eGDR by each formula in this study [21]. In addition, suggested eGDR cut-offs from the literature of four, six, and eight mg/kg/min [18], with numbers lower than these values being regarded as insulin resistant, have also been evaluated in relationship to incident CAD. The metabolic syndrome, as a categorical variable was as defined according to the Joint Statement criteria [7].

### Coronary artery disease (CAD)

CAD events (N = 539) were identified for all participants until the end of 2020 from the Finnish Care Register for Health Care, Finnish Institute for Health and Welfare, and from the death registry, Statistics Finland. CAD was defined as first coronary artery disease event of acute myocardial infarction (ICD (international classification of diseases)-8/9: 410 and 412; ICD-10: I21–23), coronary revascularisation (NOMESCO (Nordic Medico-Statistical Committee) Classification of Surgical Procedures: codes FNA (Connection to coronary artery from internal mammary artery), FNB (Connection to coronary artery from gastroepiploic artery), FNC (Aorto-coronary venous bypass), FND (Aorto-coronary bypass using prosthetic graft), FNE (Coronary bypass using free arterial graft), FNF (Coronary thromboendarterectomy), FNG (Expansion and recanalisation of coronary artery), TFN40 (Catherisation of heart with balloon widening of coronary vessels), FN1AT (Endovascular dilatation of coronary arteries), FN1BT (Extensive endovascular dilatation of coronary arteries), FN1YT (Percutaneous insertion of coronary artery stent), and FN2 (Other procedures on coronary arteries)), or CAD as immediate or underlying cause of death (ICD-9: 410–414; ICD-10: 120–I25). Individuals were followed up for at least half a year until their first CAD event, death, or until the end of 2020 for a median of 19.3 years (IQR 14.6, 21.4) years, in total 59,501 person-years of follow-up.

P-values for normally distributed continuous variables were calculated using a t-test, for non-normally distributed continuous variables a Mann–Whitney test, and for categorical variables a χ^2^ test. P-values to compare more than two groups were calculated using a Kruskal–Wallis test. Correlations between eGDR scores were estimated by Spearman correlation coefficients. Kaplan–Meier survival curves were constructed by quartiles of all eGDR scores and for the presence of the metabolic syndrome. Time-to-event analyses with CAD as outcome were performed using Cox proportional hazard regression models [22]. The proportional hazard assumption was tested based on Schoenfeld residuals and if violated we restricted the follow-up to 15 years as the hazards were proportional up to that time. As the different formulae use different variables to calculate eGDR, we did not adjust the analyses with further covariates to ensure a fair comparison. As the cohort was selected in a way to avoid any missing information, there was no need to address missing information. When eGDR scores were modelled as a continuous variable, we tested for linearity using the Wald test and if indicated modelled the relationship with cubic splines. Martingale residuals were assessed, and variables were log transformed when necessary. The performance of the three formulae and all their components on predicting CAD were compared using the Harrell C-Index. The analyses were repeated on three subsets of individuals with varying KDIGO status: low, moderate and those with a high or a very high status combined in one set. All analyses were performed in R (R Core Team version 4.2.2, Vienna, Austria).

## Results

### Baseline characteristics

Demographics are provided in Table 1, including a subdivision by subsequent CAD status. From 3517 FinnDiane Study participants, 539 experienced a CAD event during the 19-year follow-up. At baseline those who subsequently developed CAD vs. those who did not, were older, had longer diabetes duration, higher BP, higher BMI and waist height ratio, higher triglycerides, total and LDL cholesterol concentrations, lower HDL cholesterol concentrations, and were more likely to be in the KDIGO category high or very high. Additionally, they were more likely to have the metabolic syndrome, lower eGDR scores, to be on antihypertensive and lipid-lowering drugs, and more likely to have a history of smoking. Insulin pumps were used by 3.5 vs. 6.3% of those who did vs. did not develop CAD respectively (p = 0.02).

**Table 1:** Baseline clinical characteristics with and without incident coronary artery disease during follow-up

	Missing *N*	No incident CAD *N* = *2978*	Incident CAD *N* = *539*	p-value
Men, %	–	48.46	51.39	0.227
Age, years	–	35.21 (10.89)	44.57 (10.79)	< 0.001
Diabetes duration, years	–	19.00 (11.16)	29.13 (10.97)	< 0.001
Onset age, years	–	16.20 (9.33)	15.43 (8.93)	0.076
Onset before 10 years of age, %	–	28.48	31.54	0.164
Onset before 5 years of age, %	–	9.70	11.50	0.229
HbA_1c_, %	–	8.33 (1.45)	8.80 (1.41)	< 0.001
HbA_1c_, mmol/mol	–	67.55 (15.88)	72.65 (15.4)	< 0.001
HbA_1c_ below 7%, %	–	15.55	6.31	< 0.001
Insulin dose, IU/kg	–	0.69 (0.55, 0.85)	0.65 (0.52, 0.78)	< 0.001
Insulin pump use, %	1	6.28	3.53	0.016
Systolic BP, mmHg	–	130 (16)	140 (18)	< 0.001
Diastolic BP, mmHg	–	79 (9)	80 (10)	0.001
Pulse pressure, mmHg	–	51 (14)	60 (17)	< 0.001
Antihypertensive medication, %	5	26.45	57.36	< 0.001
BMI, kg/m^2^	–	25.09 (3.62)	25.79 (3.56)	< 0.001
Waist-height ratio	–	0.50 (0.06)	0.52 (0.06)	< 0.001
Waist-hip ratio	–	0.86 (0.08)	0.88 (0.09)	< 0.001
Waist circumference, cm	–	84.87 (10.97)	88.09 (11.87)	< 0.001
Total cholesterol, mmol/L	–	4.82 (0.91)	5.29 (1.00)	< 0.001
LDL cholesterol, mmol/L	38	2.90 (0.83)	3.35 (0.87)	< 0.001
HDL cholesterol, mmol/L	–	1.39 (0.39)	1.29 (0.39)	< 0.001
Non-HDL cholesterol, mmol/L	–	3.43 (0.95)	4.00 (1.06)	< 0.001
Triglycerides, mmol/L	–	0.96 (0.73, 1.36)	1.12 (0.85, 1.61)	< 0.001
Lipid-lowering medication, %	6	6.86	18.55	< 0.001
Metabolic syndrome, %	7	34.29	57.06	< 0.001
History of smoking, %	131	42.98	51.46	< 0.001
KDIGO				
Low, %	–	76.19	45.27	< 0.001
Moderate, %	–	13.87	19.11	0.002
High, %	–	5.47	16.33	< 0.001
very high, %	–	4.47	19.29	< 0.001
eGDR				
Williams, mg/kg/min	–	7.43 (2.24)	5.78 (2.42)	< 0.001
Duca, mg/kg/min	–	3.8 (1.46)	3.53 (1.43)	< 0.001
Januszewski, mg/kg/min	–	6.55 (3.75, 9.17)	4.09 (0.12, 7.15)	< 0.001

Data are mean (SD), median (IQR), or percentages. BP: blood pressure; KDIGO: Kidney Disease Improving Global Outcomes risk categories; eGDR: estimated glucose disposal rate

### Comparison of insulin resistance by eGDR formulae’s lowest quartile and metabolic syndrome

There was little overlap in the number of insulin-resistant individuals when defined as being in the lowest quartile in each of the eGDR formulae (Additional file 1: Fig. S1). The frequency of the metabolic syndrome in participants considered insulin-resistant, i.e., the lowest quartile of eGDR by the Williams, Duca, and Januszewski eGDR formulae, was 64, 71, and 64%, respectively. In comparison, for those in the highest quartile of eGDR, the corresponding frequencies were 17, 16, and 17%.

As a continuous score calculated with the three assessed formulae, the eGDRs were significantly (p < 0.001), albeit weakly, correlated: The Spearman correlation coefficient was 0.42 for Williams vs. Duca scores; 0.10 for Williams vs. Januszewski; and 0.10 for Duca vs. Januszewski.

### eGDR, the metabolic syndrome, and KDIGO risk categories

Baseline characteristics by KDIGO categories are provided in Additional file 1: Table S2. Worsening kidney disease was associated with higher rates of CAD, male sex, longer diabetes duration, younger age of diabetes onset, higher HbA_1c_ concentrations, and worse traditional risk factors, such as adiposity, BP, and lipids. For all three formulae, the eGDR decreased with worsening KDIGO risk category, and, in addition, the percentage of individuals with metabolic syndrome increased with higher KDIGO category.

### Associations between baseline eGDR quartiles and subsequent CAD

Kaplan–Meier curves for incidence of CAD (Additional file 1: Fig. S2) by eGDR quartiles showed increasing separation of curves over follow-up time, with different patterns of spread between formulae, reaching statistical significance for all eGDR formulae: eGDR by Williams and Januszewski, both p < 0.001, and eGDR by Duca, p = 0.015. Similarly, metabolic syndrome status curves separated significantly (p < 0.001) over time, with higher CAD rates in those with vs. without the metabolic syndrome at baseline (Additional file 1: Fig. S3).

### Risk for CAD based on baseline eGDR score and by kidney disease severity

**Fig. 1:**
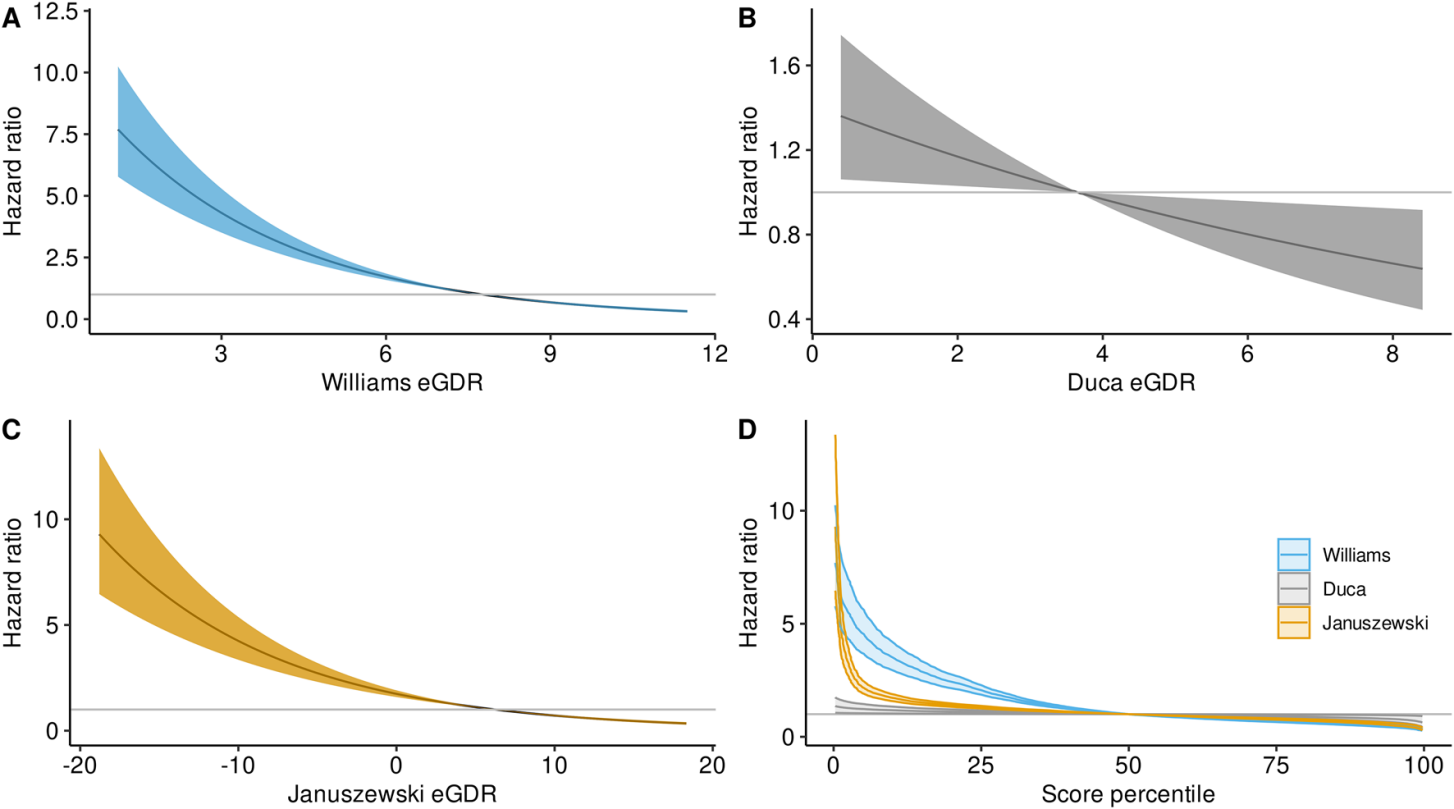
Cohort-wide hazard ratios for incident coronary artery disease by estimated glucose disposal rate formulae. Williams (**A**), Duca (**B**), Januszewski (**C**). **D** Compares all three formulae and shows the hazard ratio per score percentile

At all the proposed cut-offs for eGDR calculated by the Williams and Januszewski formulae, we found an association with the CAD incidence (Table 2). The strengths of the associations varied depending on the cut-off, but was stronger for scores based on the Williams formula at all cut-offs. For the Duca formula, only the lowest quartile of its eGDR was associated with incidence of CAD. When restricting the follow-up time to maximum of 15 years, the hazard ratio (HR) for CAD incidence decreased linearly with an increasing eGDR score for all three formulae (Fig. 1A–C), indicating that any increase in eGDR (improvement in insulin sensitivity) was cardioprotective. In Fig. 1D, the HRs are defined per score percentile and therefore allow for a direct comparison of the strength of the association for all three formulae. When using the C-index, the Williams-derived eGDR discriminated individuals with regards to CAD incidence either better or at least equally well compared to the other formulae. Only in the lowest score percentiles, the HRs based on Januszewski were higher, but their 95% confidence intervals (CI) overlapped with the Williams formula, e.g., in the 0.28 percentile Januszewski-based HR was 9.29 [6.46, 13.36], whereas the Williams-based HR was 7.69 [5.78, 10.24].

**Table 2:** Hazard ratios and 95% confidence intervals for incident coronary artery disease in the full cohort

	Lowest score quartile Q1 vs. Q2–4 (ref)	Score < 4 vs. score ≥ 4 (ref)	Score < 6 vs. score ≥ 6 (ref)	Score < 8 vs. score ≥ 8 (ref)
eGDR Williams	3.61 [3.05;4.28] p < 0.001	3.08 [2.54;3.74] p < 0.001	3.97^†^ [3.19;4.94] p < 0.001	3.18 [2.61;3.89] p < 0.001
eGDR Duca	1.34 [1.11;1.61] p = 0.002	1.09 [0.92;1.30] p = 0.33	1.41 [0.95;2.11] p = 0.09	2.72 [0.38;19.32] p = 0.32
eGDR Januszewksi	2.09 [1.76;2.48] p < 0.001	2.54^†^ [2.06;3.15] p < 0.001	2.13^†^ [1.70;2.66] p < 0.001	1.61 [1.31;1.98] p < 0.001

^†^As the proportional hazard assumption was violated for the full follow-up time, the follow-up time was restricted to a maximum of 15 years

Additional file 1: Figures S4, S5, and S6 show the HR for the 15 years incidence of CAD, separately for each KDIGO category. In all three categories, when using the eGDR score by Williams, the HR for CAD decreased linearly with increasing insulin sensitivity.

### Comparison of baseline eGDR scores with other risk factors for subsequent CAD

**Fig. 2:**
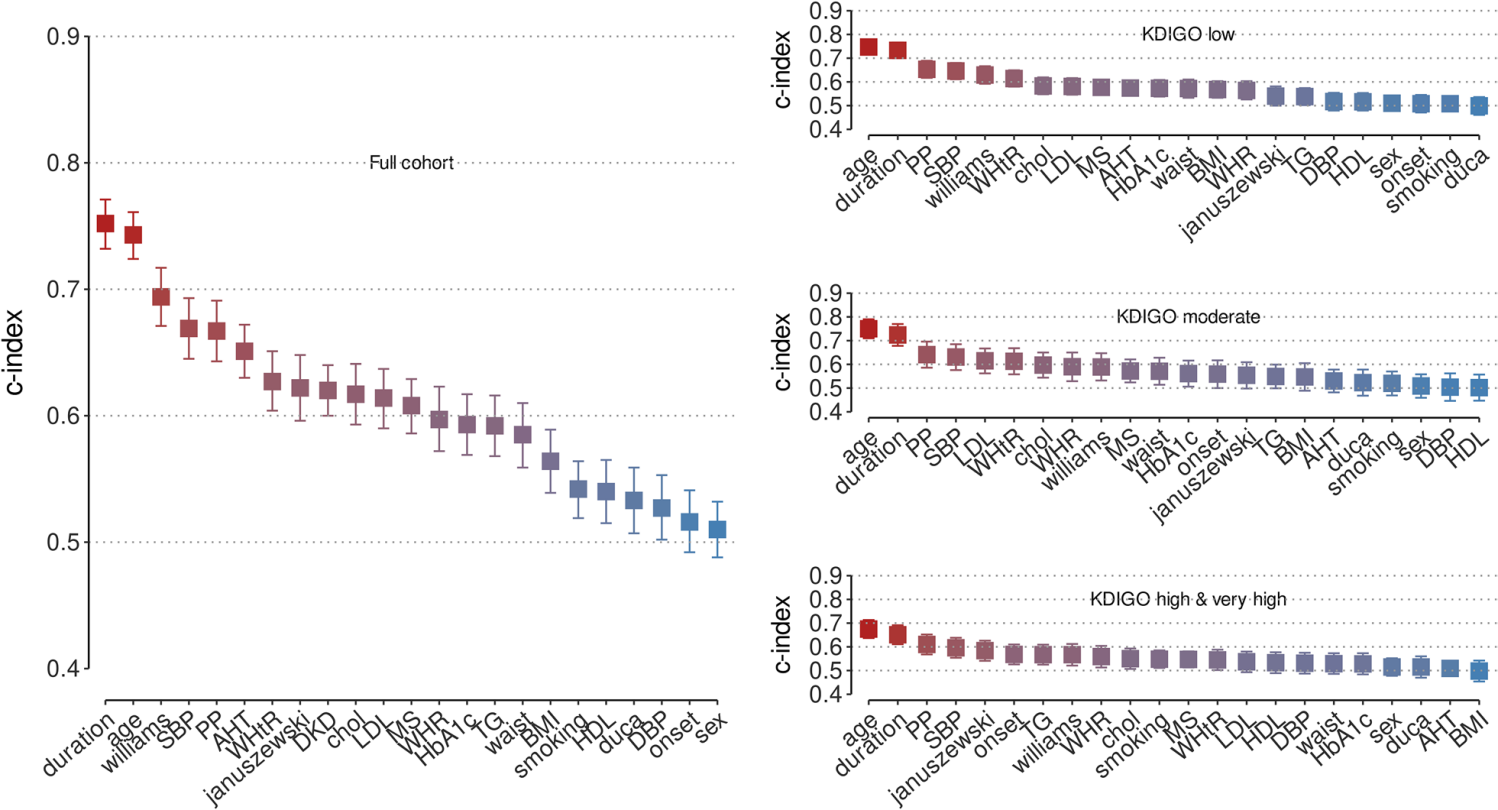
C-indexes for cardiovascular risk factors including estimated glucose disposal rate scores. For all individuals and separately for individuals in Kidney Disease Improving Global Outcomes risk categories low, moderate and high combined with very high

As shown in Fig. 2 and Additional file 1: Tables S3–S6, age and diabetes duration showed the highest C-index for the association with incident CAD for the whole cohort and for each KDIGO risk category. In the full cohort, the eGDR score by Williams had a higher C-index compared to the Januszewski score (0.69, 95% CI [0.67, 0.72] vs. 0.62 [0.60, 0.65]) and the Duca score (0.53 [0.51, 0.56]). This was observed in the low KDIGO category (Additional file 1: Table S4), however in the moderate (Additional file 1: Table S5) as well as the pooled high and very high KDIGO categories (Additional file 1: Table S6), there were no significant differences between the eGDR scores among the three formulae as the 95% CIs overlapped. However, in the KDIGO categories high and very high, the C-index for Januszewski was nominally higher than the C-index for Williams, but the CIs overlapped (0.58, [0.54, 0.63] vs. 0.57 [0.52, 0.61]).

## Discussion

In the present study, we compared the performance of three different eGDR formulae with respect to their ability to predict subsequent incident CAD at different stages of kidney disease severity in people with type 1 diabetes. We found that correlations between the three assessed formulae were weak keeping with findings from a cross-sectional clamp study series [11]. Importantly, eGDR formulae are derived from different individuals, may have had variations in clamp methodology, and include different factors with different weightings. It is also recognised that eGDR likely reflects other processes, which modulate the insulin sensitivity. However, despite the weak correlations between the eGDR formulae, the lowest eGDR quartiles of all formulae, which represents the individuals with insulin resistance, were associated with higher risk of CAD. This was true also for the metabolic syndrome, in line with previous studies [23]. Regarding the group with the lowest quartile of eGDR, the overlapping number of subjects for two or three formulae was low, indicating that the different formulae identify different individuals with lower insulin sensitivity, which also makes comparison of the formulae’s risk prediction performance relevant.

Whilst both measured and estimated insulin sensitivity may vary with kidney disease severity [2, 24], clamp studies usually exclude people with moderate or severe kidney disease, and eGDR formulae specific for type 1 diabetic kidney disease have not been developed, nor have existent formulae been systematically assessed at different stages of kidney disease, although kidney disease is a major predictor of CAD. Therefore, it is an important and novel finding that the performance of the three eGDR formulae for subsequent CAD events varied by eGDR formulae and KDIGO risk category.

Nonetheless, the Williams eGDR score was the strongest (based on C-index) predictor of CAD overall. Only the non-modifiable risk factors age and diabetes duration were stronger predictors than eGDR by Williams. Some differences in the Williams, Duca, and Januszewski clamp cohorts could potentially explain the different abilities of their respective eGDR formulae to predict CAD (Additional file 1: Table S7). The individuals in the Duca cohort were somewhat older compared to the Williams cohort (mean age 45.6 vs. 35.9 years), but the Williams cohort had a higher HbA1c (9.5%) compared both to the Duca cohort (7.6%) as well as the Januszewski cohort (7.7%). As both age and glycaemic control are independent and important risk factors for CAD, the cohorts are at different risks of developing CAD to begin with, which might also influence the models’ different abilities to predict CAD. It is, however, worth noting that although the characteristics differ between the FinnDiane participants and the Williams cohort, the eGDR by Williams was a strong predictor of CAD.

Particularly with the increasing global incidence and prevalence rates of type 1 diabetes [25] and also of obesity in people with type 1 diabetes, eGDR formulae may be useful as a surrogate endpoint in clinical research and clinical practice. In the full cohort, the Williams eGDR score ranked higher than its components, but systolic BP and pulse pressure predicted CAD equally well. In the moderate KDIGO category, the components of the Williams formula performed equally well, but waist-height ratio performed nominally better than the Williams score. In the KDIGO low category, pulse pressure, a component of the Januszewski formula, outperformed the score, and in all other settings, pulse pressure performed at least equally well. In addition, the Januszewski formula also includes serum creatinine, which possibly explains its performance in the KDIGO high and very high cohort. In the overall cohort, the continuous Williams eGDR score was a better predictor of CAD than the dichotomous metabolic syndrome score. Of note, the waist-height ratio which is a marker of central fatness and is linked to insulin sensitivity, performed similar to the presence of the metabolic syndrome for the prediction of CAD in the entire cohort and in all KDIGO categories. This finding highlights the relevance of central obesity in the metabolic syndrome and is also aligned with a previous publication showing that the waist-height ratio is associated with visceral fat mass independent of sex and kidney disease status in adults with type 1 diabetes [26].

Importantly, only the eGDR by Williams showed a linear association with CAD risk in all (sub-) analyses. Therefore, eGDR based on Williams would lend itself well to evaluating the effects of interventions, which may improve insulin sensitivity and might be a better choice than a categorical variable such as the metabolic syndrome. Potential interventions are weight-loss, exercise and muscle gain, insulin sensitiser drugs (e.g., metformin, sodium-glucose cotransporter-2 (SGLT2) inhibitors, and incretin-based drugs), and the use of insulin pumps. There are past and ongoing trials of adjunct therapy in people with type 1 diabetes and large real-world databases, which could be used to test the facility of eGDR scores in clinical trials and in clinical practice.

Study strengths include the large, observational ‘real-world’ FinnDiane cohort with detailed characterisation of participants, long and ongoing follow-up, moderately high rates of CAD, and wide range of kidney status. Limitations are that not all existent insulin sensitivity estimating formulae have been evaluated [27]. However, importantly, the eGDR formulae are derived from a small number of clamp studies. We included one formula each from the clamp studies from Williams, Duca, and Januszewski, and excluded the clamp studies from youth and non-Caucasian populations. Furthermore, this small number of clamp studies included only few people with kidney disease and were performed in different populations, limiting the generalisability of eGDR formulae for other ages, ethnicities, and body habitus. The original formula by Duca (and also another formula by Januszewski) included serum adiponectin, which associates strongly with insulin sensitivity. Due to limited data on adiponectin, for this study we selected the best fit formula without adiponectin, which might have impacted on the weaker results observed for the Duca eGDR formula. There is uncertainty as to the effects of different types of lifestyles, drugs, and insulin delivery modality on eGDR. Furthermore, CAD may be silent in people with diabetes, which if anything would dilute our data.

## Conclusions

Scoring adults with type 1 diabetes based on three formulae to estimate insulin sensitivity matters, as the lowest quartile of each score was associated with CAD. While some individual components of the eGDR formulae performed better than the eGDR score in predicting incident CAD, an eGDR score provides insight to insulin sensitivity, beyond CAD risk estimation, and offers a broader risk score that could succinctly evaluate treatment or lifestyle interventions. Notably, the strength of association varies by formula and kidney disease status. As a continuous measure to assess CAD risk, the Williams eGDR score appears particularly promising due to its linear association that is independent of kidney disease subclass.

### Supplementary Information

**Additional file 1:** **Table S1.** Physicians and nurses at each of the FinnDiane centres participating in patient recruitment and characterisation. **Table S2.** Baseline clinical characteristics at according to the Kidney Disease Improving Global Outcomes (KDIGO) risk categories. **Table S3.** C-indexes and 95% confidence intervals (CI) with regards to coronary artery disease (CAD) for three estimated glucose disposal rate (eGDR) formulae, the metabolic syndrome and their components for the full cohort. **Table S4.** C-indexes and 95% confidence intervals (CI) with regards to coronary artery disease (CAD) for three estimated glucose disposal rate (eGDR) formulae, the metabolic syndrome and their components for individuals in Kidney Disease Improving Global Outcomes (KDIGO) category low. **Table S5.** C-indexes and 95% confidence intervals (CI) with regards to coronary artery disease (CAD) for three estimated glucose disposal rate (eGDR) formulae, the metabolic syndrome and their components for individuals in Kidney Disease Improving Global Outcomes (KDIGO) category moderate. **Table S6.** C-indexes and 95% confidence intervals (CI) with regards to coronary artery disease (CAD) for three estimated glucose disposal rate (eGDR) formulae, the metabolic syndrome and their components for individuals in Kidney Disease Improving Global Outcomes (KDIGO) categories high and very high. **Table S7**. Comparison of the FinnDiane study participants to those in the clamp studies. **Figure S1.** A Venn diagram for insulin resistance defined as those individuals that were ranked in the lowest quartile of each estimated glucose disposal rate (eGDR) score. **Figure S2.** Kaplan–Meier curves for subsequent coronary artery disease (CAD) based on baseline estimated glucose disposal rete (eGDR) quartiles for (A) eGDR by Williams; (B) eGDR by Duca, and (C) eGDR by Januszewski. **Figure S3.** Kaplan–Meier curves for subsequent coronary artery disease (CAD) based on metabolic syndrome (yes vs. no). **Figure S4.** Hazard ratio plot for coronary artery disease according to different eGDR formulae in individuals at KDIGO category low. **Figure S5.** Hazard ratio plot for coronary artery disease according to different eGDR formulae in individuals at KDIGO category moderate. **Figure S6.** Hazard ratio plot for coronary artery disease according to different eGDR formulae in individuals at KDIGO category high & very high.

## Data Availability

The datasets are not publicly available due to the consent provided by the participant at the time of data collection. The data access, which is subject to local regulations, can be obtained upon reasonable request by contacting: Maaria Puupponen (email: maaria.puupponen@helsinki.fi), Research Program Coordinator, Clinical and Molecular Metabolism (CAMM), University of Helsinki. Upon approval, analysis needs to be performed on a user-specific local server (with protected access) and requires the applicant to sign non-disclosure and secrecy agreements.

## Abbreviations

AER: Albumin excretion rate
AHT: Antihypertensive treatment
BMI: Body mass index
BP: Blood pressure
CAD: Coronary artery disease
CI: Confidence interval
C-index: Concordance index
eGFR: Estimated glomerular filtration rate
eGDR: Estimated glucose disposal rate
FN1AT: Endovascular dilatation of coronary arteries
FN1BT: Extensive endovascular dilatation of coronary arteries
FN1YT: Percutaneous insertion of coronary artery stent
FN2: Other procedures on coronary arteries
FNA: Connection to coronary artery from internal mammary artery
FNB: Connection to coronary artery from gastroepiploic artery
FNC: Aorto-coronary venous bypass
FND: Aorto-coronary bypass using prosthetic graft
FNE: Coronary bypass using free arterial graft
FNF: Coronary thromboendarterectomy
FNG: Expansion and recanalization of coronary artery
HbA1_c_: Glycated haemoglobin
HDL: High density lipoprotein
HR: Hazard ratio
ICD: International classification of diseases
IQR: Interquartile range
LDL: Low density lipoprotein
NOMESCO: Nordic Medico-Statistical Committee
KDIGO: Kidney Disease: Improving Global Outcomes
SGLT2: Sodium-glucose cotransporter-2
TFN40: Catherisation of heart with balloon widening of coronary vessels
WHR: Waist-to-hip ratio
