# Supplementary material for: Insulin sensitivity estimates and their longitudinal association with coronary artery disease in type 1 diabetes. Does it matter?
Source: Cardiovasc Diabetol. 2024 May 3;23:152. doi: 10.1186/s12933-024-02234-x (PMC11069169; doi:10.1186/s12933-024-02234-x)

**Additional file:**

**Insulin sensitivity estimates and their association with coronary artery disease in type 1 diabetes. Does it matter?**

Stefan Mutter, PhD^1,2,3^, Erika B Parente, PhD^1,2,3^, Andrzej S Januszewski, PhD^4,5^, Johan R Simonsen, DMSc^1,2,3^, Valma Harjutsalo, PhD^1,2,3^, Per-Henrik Groop, DMSc, FRCPE^1,2,3,6,7^, Alicia J Jenkins, MD, FRACP*^5,7^, Lena M Thorn*, DMSc^1,3,8^, on behalf of the FinnDiane Study Group

*equal contributions

¹*Folkhälsan Institute of Genetics, Folkhälsan Research Center, Biomedicum Helsinki, Haartmaninkatu 8, 00290 Helsinki, Finland*

*²Department of Nephrology, University of Helsinki and Helsinki University Hospital, Haartmaninkatu 4, 00290 Helsinki, Finland*

³*Research Program for Clinical and Molecular Metabolism, Faculty of Medicine, University of Helsinki, Haartmaninkatu 8, 00290 Helsinki, Finland*

*^4^Sydney Pharmacy School, University of Sydney, A15, Science Rd, Camperdown NSW 2050, Australia*

*^5^NHMRC Clinical Trials Centre, University of Sydney, K25, Parramatta Rd, Camperdown NSW 2050, Australia*

*^6^Department of Diabetes, Central Clinical School, Monash University, The Alfred Centre*

*99 Commercial Rd, Melbourne VIC 3004, Australia*

*^7^Baker Heart and Diabetes Institute, 75 Commercial Rd, Melbourne VIC 3004, Australia*

*^8^Department of General Practice and Primary Health Care, University of Helsinki and Helsinki University Hospital, Biomedicum 2, Tukholmankatu 8, 00290 Helsinki, Finland*

Table of Contents

[Table S1. Physicians and nurses at each of the FinnDiane centres participating in patient recruitment and characterisation. 4](#_Toc163474796)

[Table S2. Baseline clinical characteristics at according to the Kidney Disease Improving Global Outcomes (KDIGO) risk categories. 6](#_Toc163474797)

[Table S3. C-indexes and 95% confidence intervals (CI) with regards to coronary artery disease (CAD) for three estimated glucose disposal rate (eGDR) formulae, the metabolic syndrome and their components for the full cohort. 7](#_Toc163474798)

[Table S4. C-indexes and 95% confidence intervals (CI) with regards to coronary artery disease (CAD) for three estimated glucose disposal rate (eGDR) formulae, the metabolic syndrome and their components for individuals in Kidney Disease Improving Global Outcomes (KDIGO) category low. 8](#_Toc163474799)

[Table S5. C-indexes and 95% confidence intervals (CI) with regards to coronary artery disease (CAD) for three estimated glucose disposal rate (eGDR) formulae, the metabolic syndrome and their components for individuals in Kidney Disease Improving Global Outcomes (KDIGO) category moderate. 9](#_Toc163474800)

[Table S6. C-indexes and 95% confidence intervals (CI) with regards to coronary artery disease (CAD) for three estimated glucose disposal rate (eGDR) formulae, the metabolic syndrome and their components for individuals in Kidney Disease Improving Global Outcomes (KDIGO) categories high and very high. 10](#_Toc163474801)

[Table S7. Comparison of the FinnDiane study participants to those in the clamp studies 11](#_Toc163474802)

[Figure S1. A Venn diagram for insulin resistance defined as those individuals that were ranked in the lowest quartile of each estimated glucose disposal rate (eGDR) score. 12](#_Toc163474803)

[Figure S2. Kaplan-Meier curves for subsequent coronary artery disease (CAD) based on baseline estimated glucose disposal rete (eGDR) quartiles for (A) eGDR by Williams; (B) eGDR by Duca, and (C) eGDR by Januszewski 13](#_Toc163474804)

[Figure S3. Kaplan-Meier curves for subsequent coronary artery disease (CAD) based on metabolic syndrome (yes vs. no). 14](#_Toc163474805)

[Figure S4. Hazard ratio plot for coronary artery disease according to different eGDR formulae in individuals at KDIGO category low. 15](#_Toc163474806)

[Figure S5. Hazard ratio plot for coronary artery disease according to different eGDR formulae in individuals at KDIGO category moderate 16](#_Toc163474807)

[Figure S6. Hazard ratio plot for coronary artery disease according to different eGDR formulae in individuals at KDIGO category high & very high. 17](#_Toc163474808)

# Table S1. Physicians and nurses at each of the FinnDiane centres participating in patient recruitment and characterisation.

| **The Finnish Diabetic Nephropathy Study Center** | **Physicians and nurses** |
| --- | --- |
| Anjalankoski Health Center | S.Koivula, T.Uggeldahl |
| Central Finland Central Hospital, Jyväskylä | T.Forslund, A.Halonen, A.Koistinen, P.Koskiaho, M.Laukkanen, J.Saltevo, M.Tiihonen |
| Central Hospital of Åland Islands, Mariehamn | M.Forsen, H.Granlund, A.-C.Jonsson, B.Nyroos |
| Central Hospital of Kanta-Häme, Hämeenlinna | P.Kinnunen, A.Orvola, T.Salonen, A.Vähänen |
| Central Hospital of Kymenlaakso, Kotka | R.Paldanius, M.Riihelä, L.Ryysy |
| Central Hospital of Länsi-Pohja, Kemi | H.Laukkanen, P.Nyländen, A.Sademies |
| Central Ostrobothnian Hospital District, Kokkola | S.Anderson, B.Asplund, U.Byskata, P.Liedes, M.Kuusela, T.Virkkala |
| City of Espoo Health Centers | A.Lyytinen, A.Nikkola, M.Niska, E.Oukko-Ruponen, E.Ritola, H.Saarinen, T.Virtanen |
| City of Helsinki Health Centers | J.Haaga, A.Kaprio, H.Kari, J.Kärkkäinen, P.Kääriäinen, A-L.Pietiläinen, B.Rantaeskola, T.Simonen |
| City of Hyvinkää Health Center | S.Klemetti, T.Nyandoto, E.Rontu, S.Satuli-Autere |
| City of Vantaa Health Centers | R.Ahonen, A.Airas, M.Erola, M.Ivaska-Suomela, E.Jatkola, A.Jauhiainen, J.Laakso, M.Laine, R.Lönnblad, A.Malm, J.Mäkelä, T.Pellonpää, R.Puranen, E.Rautamo, K.Rautavaara, R.Toivonen, H.Virtanen |
| Heinola Health Center | P.Hentunen, J.Lagerstam |
| Helsinki University Central Hospital, Department of Medicine, Division of Nephrology | A. Dufva, M.Feodoroff, D.Gordin, O.Heikkilä, K.Hietala, J.Fagerudd, M.Korolainen, L.Kyllönen, J.Kytö, S.Lindh, K.Pettersson-Fernholm, M.Rosengård-Bärlund, A.Sandelin, L.Thorn, J.Tuomikangas, K. Uljala, T.Vesisenaho, J.Wadén |
| Herttoniemi Hospital, Helsinki | V.Sipilä |
| Hospital of Lounais-Häme, Forssa | T.Kalliomäki, J.Koskelainen, R.Nikkanen, N.Savolainen, H.Sulonen, E.Valtonen |
| Hyvinkää Hospital | L. Norvio, A.Hämäläinen |
| Iisalmi Hospital | E.Toivanen |
| Jokilaakso Hospital, Jämsä | A.Parta, I.Pirttiniemi |
| Jorvi Hospital, Helsinki University Central Hospital | S.Aranko, S.Ervasti, R.Kauppinen-Mäkelin, A.Kuusisto, T.Leppälä, K.Nikkilä, L.Pekkonen |
| Jyväskylä Health Center, Kyllö | K.Nuorva, M.Tiihonen |
| Kainuu Central Hospital, Kajaani | S.Jokelainen, K.Kananen, M.Karjalainen, P.Kemppainen, A-M.Mankinen, A.Reponen, M.Sankari |
| Kerava Health Center | H.Stuckey, P.Suominen |
| Kirkkonummi Health Center | A.Lappalainen, M.Liimatainen, J.Santaholma |
| Kivelä Hospital, Helsinki | A.Aimolahti, E.Huovinen |
| Koskela Hospital, Helsinki | V.Ilkka, M.Lehtimäki |
| Kotka Health Center | E.Pälikkö-Kontinen, A.Vanhanen |
| Kouvola Health Center | E.Koskinen, T.Siitonen |
| Kuopio University Hospital | E.Huttunen, R.Ikäheimo, P.Karhapää, P.Kekäläinen, M.Laakso, T.Lakka, E.Lampainen, L.Moilanen, S. Tanskanen, L.Niskanen, U.Tuovinen, I.Vauhkonen, E.Voutilainen |
| Kuusamo Health Center | T.Kääriäinen, E.Isopoussu |
| Kuusankoski Hospital | E.Kilkki, I.Koskinen, L.Riihelä |
| Laakso Hospital, Helsinki | T.Meriläinen, P.Poukka, R.Savolainen, N.Uhlenius |
| Lahti City Hospital | A.Mäkelä, M.Tanner |
| Lapland Central Hospital, Rovaniemi | L.Hyvärinen, K.Lampela, S.Pöykkö, T.Rompasaari, S.Severinkangas, T.Tulokas |
| Lappeenranta Health Center | P. Erola, L.Härkönen, P.Linkola, T.Pekkanen, I.Pulli, E.Repo |
| Lohja Hospital | T.Granlund, K.Hietanen, M.Porrassalmi, M.Saari, T.Salonen, M.Tiikkainen |
| Länsi-Uusimaa Hospital, Tammisaari | I.-M.Jousmaa, J.Rinne |
| Loimaa Health Center | A.Mäkelä, P.Eloranta |
| Malmi Hospital, Helsinki | H.Lanki, S.Moilanen, M.Tilly-Kiesi |
| Mikkeli Central Hospital | A.Gynther, R.Manninen, P.Nironen, M.Salminen, T.Vänttinen |
| Mänttä Regional Hospital | I.Pirttiniemi, A-M.Hänninen |
| North Karelian Hospital, Joensuu | U-M.Henttula, P.Kekäläinen, M.Pietarinen, A.Rissanen, M.Voutilainen |
| Nurmijärvi Health Center | A.Burgos, K.Urtamo |
| Oulaskangas Hospital, Oulainen | E.Jokelainen, P-L.Jylkkä, E.Kaarlela, J.Vuolaspuro |
| Oulu Health Center | L.Hiltunen, R.Häkkinen, S.Keinänen-Kiukaanniemi |
| Oulu University Hospital | R.Ikäheimo |
| Päijät-Häme Central Hospital | H.Haapamäki, A.Helanterä, S.Hämäläinen, V.Ilvesmäki, H.Miettinen |
| Palokka Health Center | P.Sopanen, L.Welling |
| Pieksämäki Hospital | V.Sevtsenko, M.Tamminen |
| Pietarsaari Hospital | M-L.Holmbäck, B.Isomaa, L.Sarelin |
| Pori City Hospital | P.Ahonen, P.Merisalo, E.Muurinen, K.Sävelä |
| Porvoo Hospital | M.Kallio, B.Rask, S.Rämö |
| Raahe Hospital | A.Holma, M.Honkala, A.Tuomivaara, R.Vainionpää |
| Rauma Hospital | K.Laine, K.Saarinen, T.Salminen |
| Riihimäki Hospital | P.Aalto, E.Immonen, L.Juurinen |
| Salo Hospital | A.Alanko, J.Lapinleimu, P.Rautio, M.Virtanen |
| Satakunta Central Hospital, Pori | M.Asola, M.Juhola, P.Kunelius, M.-L.Lahdenmäki, P.Pääkkönen, M.Rautavirta |
| Savonlinna Central Hospital | T.Pulli, P.Sallinen, M.Taskinen, E.Tolvanen, T.Tuominen, H.Valtonen, A.Vartia, S-L. Viitanen |
| Seinäjoki Central Hospital | O.Antila, E.Korpi-Hyövälti, T.Latvala, E.Leijala, T.Leikkari, M.Punkari, N.Rantamäki, H.Vähävuori |
| South Karelia Central Hospital, Lappeenranta | T.Ensala, E.Hussi, R.Härkönen, U.Nyholm, J.Toivanen |
| Tampere Health Center | A.Vaden, P.Alarotu, E.Kujansuu, H.Kirkkopelto-Jokinen, M.Helin, S.Gummerus, L.Calonius, T.Niskanen, T.Kaitala, T.Vatanen |
| Tampere University Hospital | P. Hannula, I.Ala-Houhala, R.Kannisto, T.Kuningas, P.Lampinen, M.Määttä, H.Oksala, T.Oksanen, A.Putila, H.Saha, K.Salonen, H.Tauriainen, S.Tulokas |
| Tiirismaa Health Center, Hollola | T.Kivelä, L.Petlin, L.Savolainen |
| Turku Health Center | A.Artukka, I.Hämäläinen, L.Lehtinen, E.Pyysalo, H.Virtamo, M.Viinikkala, M.Vähätalo |
| Turku University Central Hospital | K.Breitholz, R.Eskola, K.Metsärinne, U.Pietilä, P.Saarinen, R.Tuominen, S.Äyräpää |
| Vaajakoski Health Center | K.Mäkinen, P.Sopanen |
| Valkeakoski Regional Hospital | S.Ojanen, E.Valtonen, H.Ylönen, M.Rautiainen,T.Immonen |
| Vammala Regional Hospital | I.Isomäki, R.Kroneld, L.Mustaniemi, M.Tapiolinna-Mäkelä |
| Vaasa Central Hospital | S.Bergkulla, U.Hautamäki, V-A.Myllyniemi, I.Rusk |

# Table S2. Baseline clinical characteristics at according to the Kidney Disease Improving Global Outcomes (KDIGO) risk categories.

|  | KDIGO low  N = 2,513 | KDIGO moderate  N = 516 | KDIGO high & very high  N = 488 | p-value |
| --- | --- | --- | --- | --- |
| Incident CAD (%) | 9.71 | 19.96 | 39.34 | <0.001 |
| Men (%) | 46.44 | 55.23 | 54.92 | <0.001 |
| Age (years) | 35.46 (11.31) | 38.67 (11.74) | 40.56 (10.17) | <0.001 |
| Diabetes duration (years) | 18.07 (11.45) | 25.39 (11.00) | 28.27 (8.29) | <0.001 |
| Onset age (years) | 17.4 (9.42) | 13.28 (8.56) | 12.29 (7.39) | <0.001 |
| Onset before 10 years of age (%) | 23.48 | 40.50 | 44.88 | <0.001 |
| Onset before 5 years of age (%) | 7.96 | 16.28 | 13.73 | <0.001 |
| HbA1c (%) | 8.22 (1.40) | 8.73 (1.41) | 8.99 (1.56) | <0.001 |
| HbA1c (mmol/mol) | 66.35 (15.32) | 71.88 (15.38) | 74.77 (17.10) | <0.001 |
| HbA1c below 7% (%) | 16.83 | 8.14 | 6.56 | <0.001 |
| Insulin dose (IU/kg) | 0.68 (0.55, 0.84) | 0.71 (0.57, 0.90) | 0.65 (0.54, 0.79) | <0.001 |
| Pump use (%) | 5.97 | 6.20 | 4.92 | 0.622 |
| Systolic BP (mmHg) | 129 (15) | 136 (18) | 142 (19) | <0.001 |
| Diastolic BP (mmHg) | 78 (9) | 80 (10) | 82 (11) | <0.001 |
| Pulse pressure (mmHg) | 51 (13) | 56 (16) | 60 (17) | <0.001 |
| Antihypertensive medication (%) | 12.90 | 60.78 | 94.24 | <0.001 |
| BMI (kg/m^2^) | 24.95 (3.52) | 25.62 (3.62) | 26.02 (3.98) | <0.001 |
| Waist-Height ratio | 0.49 (0.06) | 0.51 (0.06) | 0.53 (0.07) | <0.001 |
| Waist-Hip ratio | 0.85 (0.08) | 0.88 (0.08) | 0.89 (0.09) | <0.001 |
| Waist (cm) | 84.24 (10.61) | 87.25 (11.56) | 89.19 (12.38) | <0.001 |
| Total cholesterol (mmol/L) | 4.77 (0.88) | 4.99 (0.91) | 5.42 (1.06) | <0.001 |
| LDL cholesterol (mmol/L) | 2.87 (0.81) | 3.05 (0.84) | 3.40 (0.93) | <0.001 |
| HDL cholesterol (mmol/L) | 1.40 (0.39) | 1.37 (0.41) | 1.26 (0.39) | <0.001 |
| Non-HDL cholesterol (mmol/L) | 3.37 (0.91) | 3.62 (0.96) | 4.15 (1.13) | <0.001 |
| Triglycerides (mmol/L) | 0.93 (0.71, 1.28) | 1.05 (0.82, 1.51) | 1.35 (0.95, 1.90) | <0.001 |
| Lipid-lowering medication (%) | 5.86 | 11.07 | 20.58 | <0.001 |
| Metabolic syndrome (%) | 30.20 | 48.16 | 65.98 | <0.001 |
| History of smoking (%) | 40.62 | 49.90 | 57.17 | <0.001 |
| *eGDR* | | | | |
| Williams (mg/kg/min) | 8.01 (1.83) | 5.83 (2.29) | 4.31 (1.74) | <0.001 |
| Duca (mg/kg/min) | 3.93 (1.46) | 3.44 (1.38) | 3.20 (1.31) | <0.001 |
| Januszewski (mg/kg/min) | 6.83 (4.35, 9.48) | 6.02 (3.07, 8.55) | 0.89 (-4.26, 5.45) | <0.001 |

Data are mean (SD), median (IQR), or percentages. CAD=coronary artery disease; BP=blood pressure; KDIGO= Kidney Disease Improving Global Outcomes risk categories; eGDR=estimated glucose disposal rate

# Table S3. C-indexes and 95% confidence intervals (CI) with regards to coronary artery disease (CAD) for three estimated glucose disposal rate (eGDR) formulae, the metabolic syndrome and their components for the full cohort.

|  | C-index [95% CI] |
| --- | --- |
| Duration | 0.75 [0.73, 0.77] |
| Age | 0.74 [0.72, 0.76] |
| Williams eGDR | 0.69 [0.67, 0.72] |
| Systolic blood pressure | 0.67 [0.64, 0.69] |
| Pulse pressure | 0.67 [0.64, 0.69] |
| Antihypertensive treatment | 0.65 [0.63, 0.67] |
| Waist-height ratio | 0.63 [0.60, 0.65] |
| Januszewski eGDR | 0.62 [0.60, 0.65] |
| Diabetic kidney disease | 0.62 [0.60, 0.64] |
| Total cholesterol | 0.62 [0.59, 0.64] |
| LDL cholesterol | 0.61 [0.59, 0.64] |
| Metabolic syndrome | 0.61 [0.59, 0.63] |
| Waist-hip ratio | 0.60 [0.57, 0.62] |
| HbA1c | 0.59 [0.57, 0.62] |
| Triglycerides | 0.59 [0.57, 0.62] |
| Waist | 0.58 [0.56, 0.61] |
| BMI | 0.56 [0.54, 0.59] |
| Smoking | 0.54 [0.52, 0.56] |
| HDL cholesterol | 0.54 [0.52, 0.56] |
| Duca eGDR | 0.53 [0.51, 0.56] |
| Diastolic blood pressure | 0.53 [0.50, 0.55] |
| Age of onset | 0.52 [0.49, 0.54] |
| Male sex | 0.51 [0.49, 0.53] |

# Table S4. C-indexes and 95% confidence intervals (CI) with regards to coronary artery disease (CAD) for three estimated glucose disposal rate (eGDR) formulae, the metabolic syndrome and their components for individuals in Kidney Disease Improving Global Outcomes (KDIGO) category low.

|  | C-index [95% CI] |
| --- | --- |
| Age | 0.75 [0.72, 0.78] |
| Duration | 0.73 [0.70, 0.77] |
| Pulse pressure | 0.65 [0.62, 0.69] |
| Systolic blood pressure | 0.65 [0.61, 0.68] |
| Williams eGDR | 0.63 [0.59, 0.67] |
| Waist-height ratio | 0.61 [0.58, 0.65] |
| Total cholesterol | 0.58 [0.55, 0.62] |
| LDL cholesterol | 0.58 [0.55, 0.62] |
| Metabolic syndrome | 0.58 [0.54, 0.61] |
| Antihypertensive treatment | 0.57 [0.55, 0.60] |
| HbA1c | 0.57 [0.54, 0.61] |
| Waist | 0.57 [0.53, 0.61] |
| BMI | 0.57 [0.53, 0.60] |
| Waist-hip ratio | 0.56 [0.53, 0.60] |
| Januszewski eGDR | 0.54 [0.50, 0.58] |
| Triglycerides | 0.54 [0.50, 0.57] |
| Diastolic blood pressure | 0.52 [0.48, 0.55] |
| HDL cholesterol | 0.52 [0.48, 0.55] |
| Male sex | 0.51 [0.48, 0.54] |
| Age of onset | 0.51 [0.47, 0.54] |
| Smoking | 0.51 [0.48, 0.54] |
| Duca eGDR | 0.50 [0.46, 0.54] |

# Table S5. C-indexes and 95% confidence intervals (CI) with regards to coronary artery disease (CAD) for three estimated glucose disposal rate (eGDR) formulae, the metabolic syndrome and their components for individuals in Kidney Disease Improving Global Outcomes (KDIGO) category moderate.

|  | C-index [95% CI] |
| --- | --- |
| Age | 0.75 [0.71, 0.79] |
| Duration | 0.72 [0.68, 0.77] |
| Pulse pressure | 0.64 [0.59, 0.70] |
| Systolic blood pressure | 0.63 [0.58, 0.69] |
| LDL cholesterol | 0.62 [0.56, 0.67] |
| Waist-height ratio | 0.61 [0.56, 0.67] |
| Total cholesterol | 0.60 [0.54, 0.65] |
| Waist-hip ratio | 0.59 [0.53, 0.65] |
| Williams eGDR | 0.59 [0.53, 0.65] |
| Metabolic syndrome | 0.57 [0.52, 0.62] |
| Waist | 0.57 [0.51, 0.63] |
| HbA1c | 0.56 [0.51, 0.62] |
| Age of onset | 0.56 [0.50, 0.62] |
| Januszewski eGDR | 0.55 [0.50, 0.61] |
| Triglycerides | 0.55 [0.50, 0.60] |
| BMI | 0.55 [0.49, 0.60] |
| Antihypertensive treatment | 0.53 [0.48, 0.58] |
| Duca eGDR | 0.52 [0.47, 0.58] |
| Smoking | 0.52 [0.47, 0.57] |
| Male sex | 0.51 [0.46, 0.56] |
| Diastolic blood pressure | 0.50 [0.45, 0.56] |
| HDL cholesterol | 0.50 [0.45, 0.56] |

# Table S6. C-indexes and 95% confidence intervals (CI) with regards to coronary artery disease (CAD) for three estimated glucose disposal rate (eGDR) formulae, the metabolic syndrome and their components for individuals in Kidney Disease Improving Global Outcomes (KDIGO) categories high and very high.

|  | C-index [95% CI] |
| --- | --- |
| Age | 0.68 [0.64, 0.71] |
| Duration | 0.65 [0.61, 0.69] |
| Pulse pressure | 0.61 [0.57, 0.65] |
| Systolic blood pressure | 0.60 [0.55, 0.64] |
| Januszewski eGDR | 0.58 [0.54, 0.63] |
| Age of onset | 0.57 [0.53, 0.61] |
| Triglycerides | 0.57 [0.52, 0.61] |
| Williams eGDR | 0.57 [0.52, 0.61] |
| Waist-hip ratio | 0.56 [0.51, 0.60] |
| Total cholesterol | 0.55 [0.51, 0.59] |
| Smoking | 0.55 [0.51, 0.58] |
| Metabolic syndrome | 0.55 [0.51, 0.58] |
| Waist-height ratio | 0.54 [0.50, 0.59] |
| LDL cholesterol | 0.54 [0.49, 0.58] |
| HDL cholesterol | 0.53 [0.49, 0.58] |
| Diastolic blood pressure | 0.53 [0.49, 0.58] |
| Waist | 0.53 [0.49, 0.57] |
| HbA1c | 0.53 [0.48, 0.57] |
| Duca eGDR | 0.52 [0.47, 0.56] |
| Male sex | 0.52 [0.48, 0.55] |
| Antihypertensive treatment | 0.51 [0.49, 0.53] |
| BMI | 0.50 [0.45, 0.54] |

# Table S7. Comparison of the FinnDiane study participants to those in the clamp studies

|  | FinnDiane | Williams | Duca | Januszewski |
| --- | --- | --- | --- | --- |
| Men (%) | 49 | 50 | 53 | 54 |
| Age (years) | 36.64 | 35.89 | 45.64 | 40.00 |
| HbA1c (%) | 8.4 | 9.5 | 7.6 | 7.7 |
| Systolic blood pressure (mmHg) | 132 | 119 | 116 | 125 |
| Diastolic blood pressure (mmHg) | 79 | 75 | 74 | 71 |
| BMI (kg/m^2^) | 25.20 | 26.73 | 26.68 | 25.20 |
| Waist hip ratio | 0.86 | 0.86 | 0.86 | 0.86 |
| Waist (cm) | 85.37 | 92.12 | 88.68 | 86.95 |
| Total cholesterol (mmol/L) | 4.89 | 4.39 | 3.59 | 4.80 |
| LDL cholesterol (mmol/L) | 2.97 | 2.66 | 1.74 | 2.90 |
| HDL cholesterol (mmol/L) | 1.38 | 1.11 | 1.48 | 1.5 |
| Triglycerides (mmol/L) | 0.98 | 3.04 | 0.81 | 0.9 |

# Figure S1. A Venn diagram for insulin resistance defined as those individuals that were ranked in the lowest quartile of each estimated glucose disposal rate (eGDR) score.


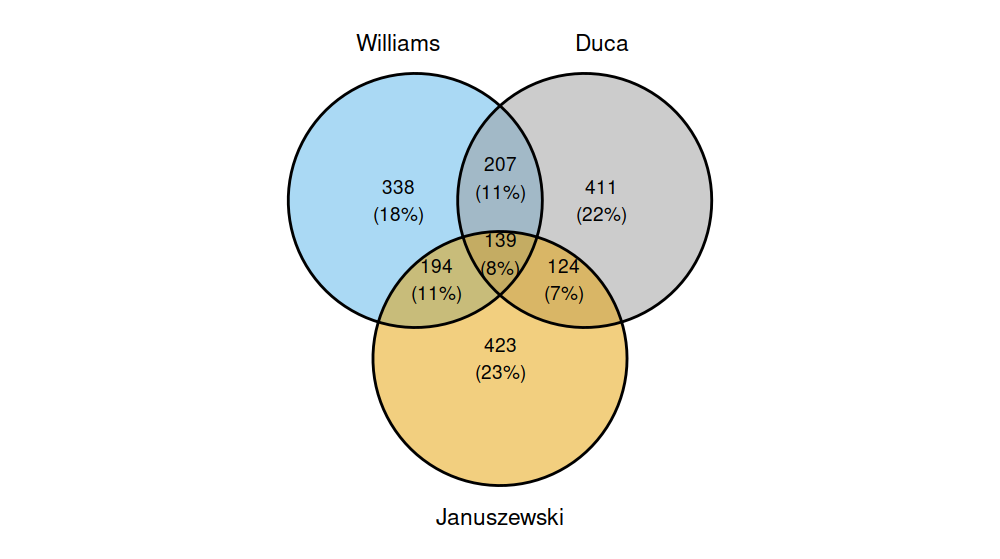


# Figure S2. Kaplan-Meier curves for subsequent coronary artery disease (CAD) based on baseline estimated glucose disposal rete (eGDR) quartiles for (A) eGDR by Williams; (B) eGDR by Duca, and (C) eGDR by Januszewski


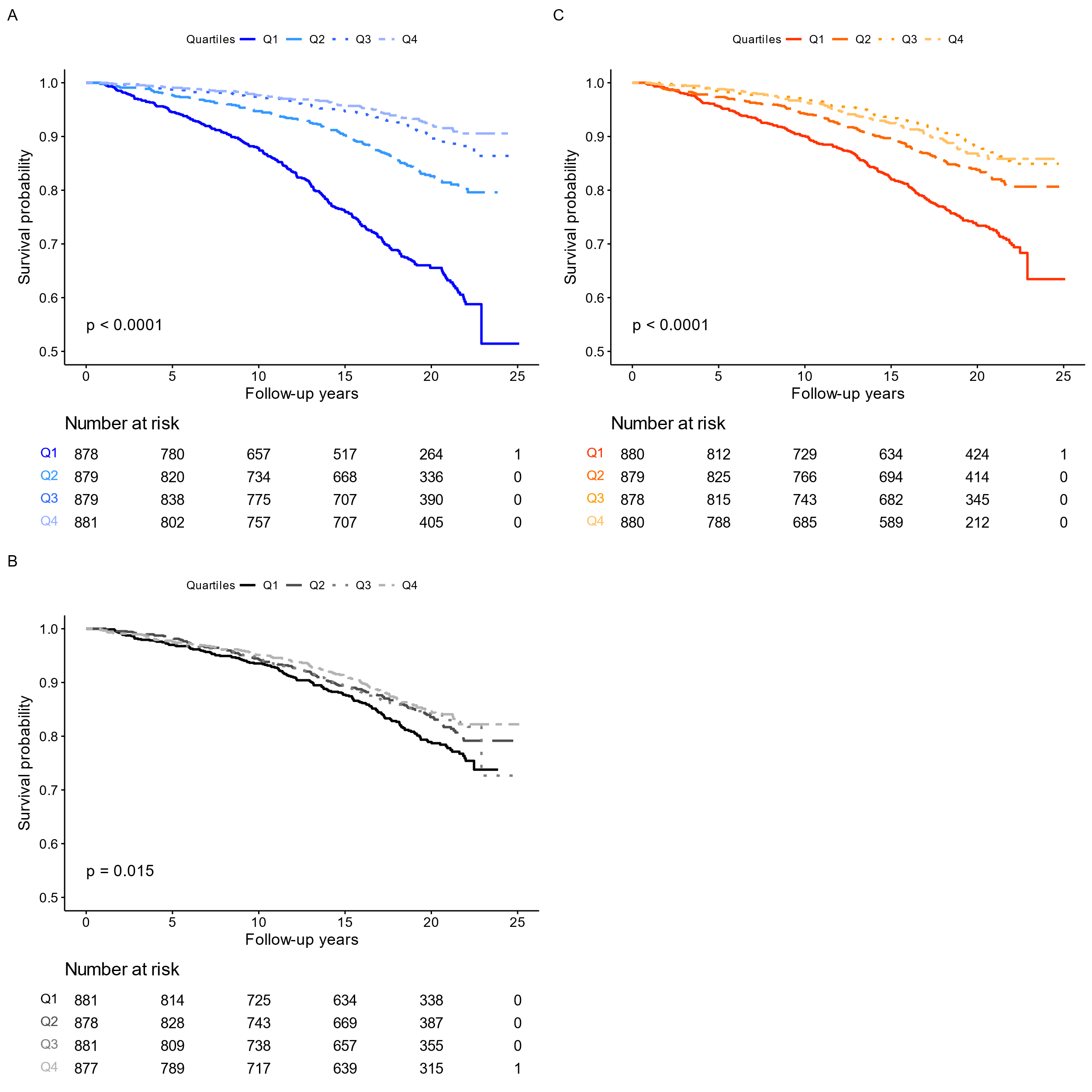


# Figure S3. Kaplan-Meier curves for subsequent coronary artery disease (CAD) based on metabolic syndrome (yes vs. no).


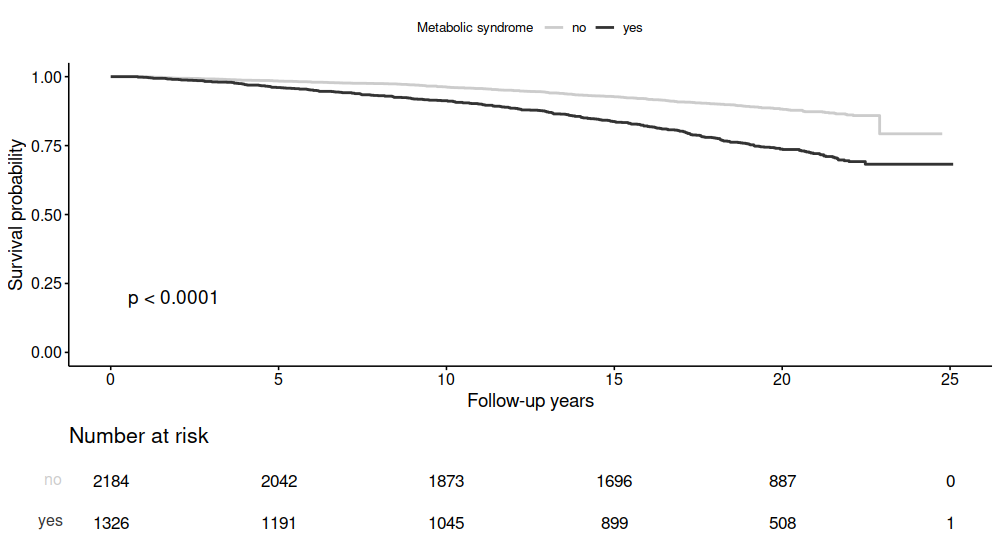


# Figure S4. Hazard ratio plot for coronary artery disease according to different eGDR formulae in individuals at KDIGO category low.


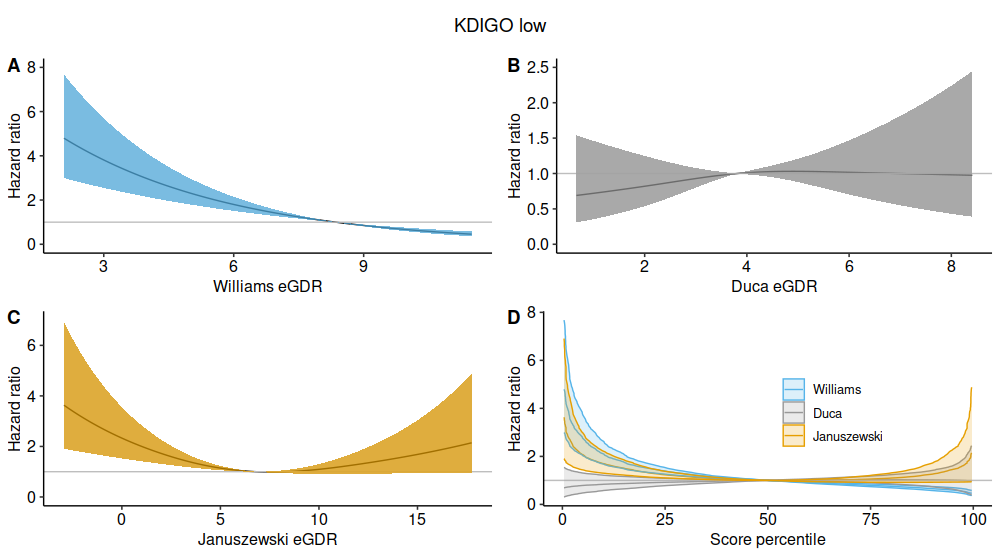


Figure S5. Hazard ratio plot for coronary artery disease according to different eGDR formulae in individuals at KDIGO category moderate**.**
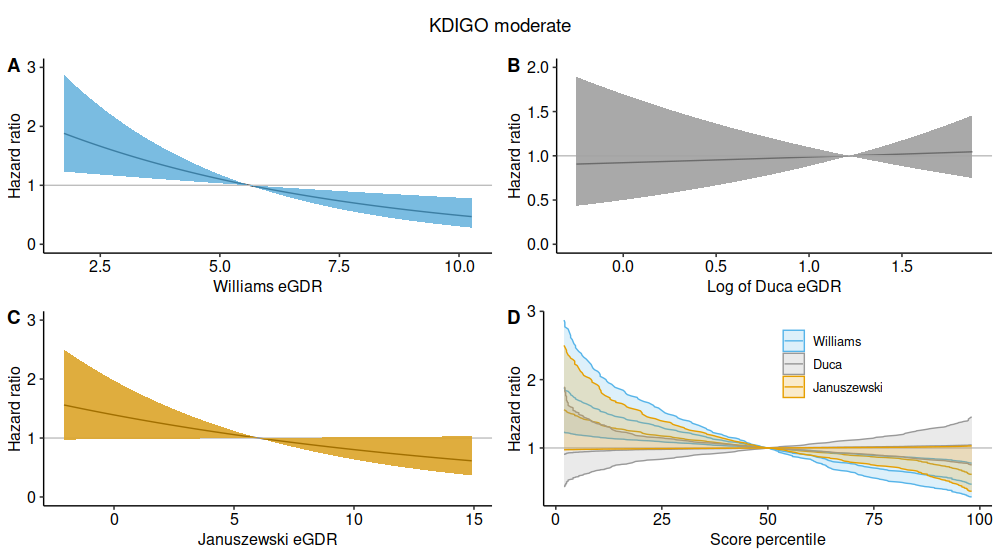


# Figure S6. Hazard ratio plot for coronary artery disease according to different eGDR formulae in individuals at KDIGO category high & very high.


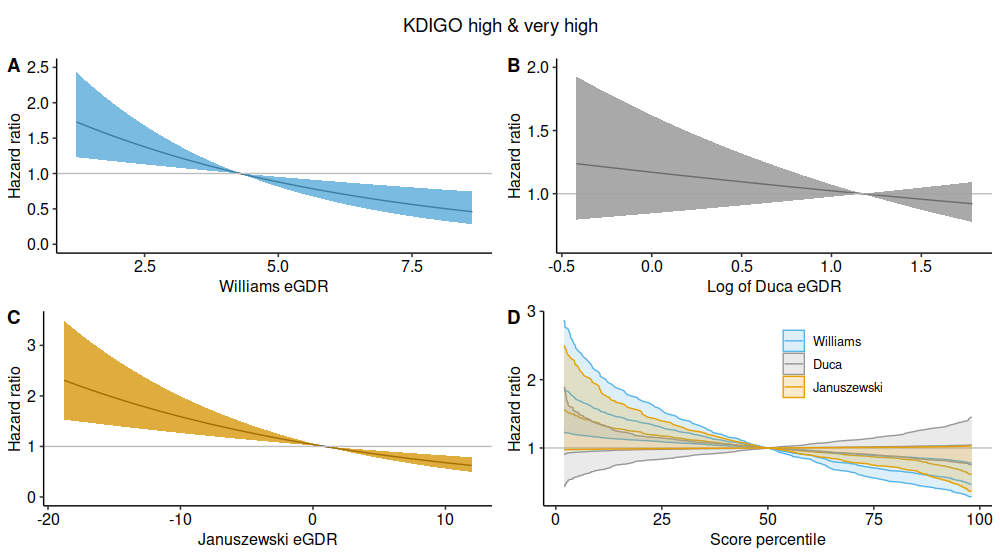

Supplement: Supplementary file 1 — Additional file 1: Table S1. Physicians and nurses at each of the FinnDiane centres participating in patient recruitment and characterisation. Table S2. Baseline clinical characteristics at according to the Kidney Disease Improving Global Outcomes (KDIGO) risk categories. Table S3. C-indexes and 95% confidence intervals (CI) with regards to coronary artery disease (CAD) for three estimated glucose disposal rate (eGDR) formulae, the metabolic syndrome and their components for the full cohort. Table S4. C-indexes and 95% confidence intervals (CI) with regards to coronary artery disease (CAD) for three estimated glucose disposal rate (eGDR) formulae, the metabolic syndrome and their components for individuals in Kidney Disease Improving Global Outcomes (KDIGO) category low. Table S5. C-indexes and 95% confidence intervals (CI) with regards to coronary artery disease (CAD) for three estimated glucose disposal rate (eGDR) formulae, the metabolic syndrome and their components for individuals in Kidney Disease Improving Global Outcomes (KDIGO) category moderate. Table S6. C-indexes and 95% confidence intervals (CI) with regards to coronary artery disease (CAD) for three estimated glucose disposal rate (eGDR) formulae, the metabolic syndrome and their components for individuals in Kidney Disease Improving Global Outcomes (KDIGO) categories high and very high. Table S7. Comparison of the FinnDiane study participants to those in the clamp studies. Figure S1. A Venn diagram for insulin resistance defined as those individuals that were ranked in the lowest quartile of each estimated glucose disposal rate (eGDR) score. Figure S2. Kaplan–Meier curves for subsequent coronary artery disease (CAD) based on baseline estimated glucose disposal rete (eGDR) quartiles for (A) eGDR by Williams; (B) eGDR by Duca, and (C) eGDR by Januszewski. Figure S3. Kaplan–Meier curves for subsequent coronary artery disease (CAD) based on metabolic syndrome (yes vs. no). Figure S4. Hazard ratio plot for [file 12933_2024_2234_MOESM1_ESM.docx]
